# Migration and dementia: a meta-analysis of epidemiological studies in Europe

**DOI:** 10.1017/S0033291720000586

**Published:** 2021-08

**Authors:** Jean-Paul Selten, Fabian Termorshuizen, Maarten van Sonsbeek, Jan Bogers, Ben Schmand

**Affiliations:** 1 Rivierduinen, Institute for Mental Health Care, Leiden, The Netherlands; 2 School for Mental Health and Neuroscience, University of Maastricht, Maastricht, The Netherlands; 3 Department of Psychology, University of Amsterdam, Amsterdam, The Netherlands; 4 Department of Medical Psychology, Amsterdam University Medical Centers, Amsterdam, The Netherlands

**Keywords:** Claudin-5, dementia, epidemiology, ethnicity, migration, social exclusion, social status

## Abstract

**Background:**

To provide an overview of epidemiological studies of dementia among migrant groups in Europe and to estimate their pooled odds ratio (OR) *v.* the reference population.

**Methods:**

Search for articles reporting on incidence or prevalence of dementia among ethnic minorities and migrants in Europe, published before 21 December 2018. We performed several meta-analyses, using a random-effects model, and, when there was no evidence of heterogeneity, a fixed-effects model. We distinguished between all migrants, African-Europeans and Asian-Europeans.

**Results:**

We retrieved five population-based surveys and two health care record studies. The latter included one incidence study, the remainder were prevalence studies. The meta-analysis of all studies yielded a pooled OR, adjusted for age and sex, of 1.73 (95% CI 1.42–2.11) for dementia in all migrant groups. However, the pooled OR of population surveys (3.10; 95% CI 2.12–4.51) was significantly higher than that for the health care record studies (OR 0.94; 95% CI 0.80–1.11). The pooled ORs for African-Europeans and Asian-Europeans, based on population surveys, were 2.54 (95% CI 1.70–3.80) and 5.36 (95% CI 2.78–10.31), respectively.

**Conclusions:**

The discrepancy between health care record studies and population surveys suggests that many migrants remain undiagnosed. Migrants from Asia and Africa seem to be at significantly increased risk of dementia in Europe. Since the prevalence rates in their countries of origin are generally not higher than those for natives in Europe, there may be a parallel with the epidemiology of schizophrenia.

## Introduction

Since the world population is ageing, age-associated diseases as dementia are expected to increase likewise. At the same time, population diversity is changing markedly, as since the 1950s emigration from several non-western countries to Europe has risen distinctly. First-generation migrants from non-western countries in Europe have now reached the age at which age-associated diseases may become manifest and several studies on the risk of dementia among migrant groups have been published.

Studies conducted in the USA concerned ethnic minorities. According to a meta-analysis, the incidence rate of Alzheimer's disease is 64% [relative risk (RR) = 1.64, 95% CI 1.35–2.00] higher among African-Americans than among Caucasian-Americans (Steenland, Goldstein, Levey, & Wharton, 2016). A systematic review, also performed in the USA, showed significantly higher incidence rates of dementia in the African-American and Caribbean-Hispanic populations than in Japanese-American, Mexican-American or non-Latino white populations (Mehta & Yeo, 2017).

There is no systematic review or meta-analysis of all European studies. A systematic review by Adelman, Blanchard, and Livingston (2009) indicated an excess of dementia among African-Caribbeans in the UK, but the magnitude of the difference and the associated risk factors remained unclear.

Consequently, the first aim of our study is to provide a systematic review of findings from incidence and prevalence studies of dementia among migrant groups and ethnic minorities in Europe. The second aim is to provide a quantitative synthesis of any difference in risk between migrants and natives (or between ethnic minority groups and the dominant population).

## Methods

The final version of the study protocol was registered with PROSPERO on 11 February 2019. PRISMA guidelines were followed (Moher, Liberati, Tetzlaff, Altman, & Prisma Group, 2009).

### Study selection

We performed an electronic search in PubMed, PsycINFO and EMBASE on 21 December 2018 to identify potentially useful articles. Key words used in the computerised search were ‘ethnic groups’, ‘emigrants and immigrants’, ‘dementia’ and ‘Europe’. Details of the search strategy are listed in online Supplementary Methods. Both free text word and related thesaurus (MeSH) terms were used.

In order to be considered for the meta-analysis, studies were required (I) to report on incidence or prevalence rate of dementia for migrant groups or ethnic minorities in Europe, with a risk ratio [RR, incidence rate ratio or odds ratio (OR)] and a 95% confidence interval (or to provide numerators and denominators for the calculation of such measures); (II) to adjust the risk ratio for differences in age (or to provide data that make this adjustment possible) and (III) to have been published in an English-language, peer-reviewed scientific journal. Reports on the frequency of cognitive decline or cognitive impairment not based on operational criteria (e.g. DSM- or ICD-criteria) and reports that failed to specify the pertinent migrant or ethnic group were excluded.

For details of the study selection, see online Supplementary Methods and Supplementary Fig. S1

### Quality check

Two authors (MvS and JPS) rated the quality of prevalence surveys independently, using the quality assessment of prevalence studies as suggested by Boyle (1998). The quality assessment was adapted with additional questions regarding the use of a screening instrument with cultural validity and a diagnosis of dementia made blind to ethnicity. We considered a diagnostic instrument valid when it had been tested in at least some of the minority ethnic groups under investigation and when the results of these tests were favourable. The possible answers were ‘yes’, ‘no’ and ‘uncertain’.

We replaced the criterion as to ‘whether special features of the sampling design were accounted for in the analysis’ by the criterion age adjustment: were the results adjusted for age differences between groups? Quality was rated using scores from 1–10, with higher numbers indicating higher quality. In case of disagreement, consensus was reached through discussion. For details of the quality assessment, see online Supplementary Table S1.

### Data extraction

Two authors (MvS and JPS) extracted the effect sizes independently. They recorded information about diagnosis (dementia and, if provided in the article, the aetiology or type of dementia), type of diagnostic criteria, ethnicity and/or country of birth, sex, adjustment for age, for sex and/or other adjustments. In case of any discrepancy, consensus was reached by discussion.

### Meta-analysis

Since the majority of the participants in the included studies were foreign-born (see below), we use the term ‘migrants’. Only one study specified subtypes of dementia (Adelman, Blanchard, Rait, Leavey, & Livingston, 2011). Consequently, our meta-analysis concerns ‘dementia, any type’.

Since there is, as yet, no evidence that ethnicity is associated with a differential survival from dementia, we felt justified to include effect estimates from both incidence and prevalence studies in our meta-analysis.

Using the available data, we created six different categories: (1) African-European, i.e. sub-Saharan African, African-Caribbean, African-Surinamese; (2) Asian-European, i.e. from the Indian subcontinent, Chinese, Asian-Surinamese; (3) White-European, i.e. migrants from the European continent; (4) from the Maghreb/Middle-East, i.e. from Morocco or Turkey; (5) other ethnicity and (6) unspecified ethnicity. The terms ‘African-Surinamese’ and ‘Asian-Surinamese’ require an explanation. The Netherlands have received many immigrants from Surinam. The population of Surinam is ethnically diverse and the two largest groups are the African-Surinamese and the Asian-Surinamese. The latter migrated in the 19^th^ century from British India to become contract-labourers on Surinamese estates.

Individuals of African ancestry were described as ‘Blacks’, ‘Black Africans’, ‘Black Caribbeans’, ‘Black Other’ or ‘Surinamese-Creole’ (i.e. African-Surinamese). Since they share a genetic background, in a first analysis we grouped them into one category. For the same reason, we added the Asian-Surinamese to those from the Indian subcontinent and China.

However, since the migration history of Africans who moved from the Caribbean or Surinam to Europe differs from that of individuals from sub-Saharan Africa, we also computed a separate effect estimate for the first group. There were too few effect estimates for the second group to allow a proper comparison between the two groups.

The age-groups examined differed by the study. Some studies published data for 5- or 10-year strata, other studies reported age-adjusted effect estimates for large age-bands (e.g. 55 years old and over). We used effect estimates adjusted for age and sex or calculated such effect estimates.

Using the information provided on numerators and denominators we calculated ORs for the migrant groups examined by Parlevliet et al. (2016). We considered the ORs adjusted for age, because of the 10-year age-strata used in the article. Since McCracken et al. (1997) provided no effect estimate, we estimated an OR using the prevalence rates and the pertinent 95% confidence intervals in index and reference populations. As no numerators or denominators for the reference group were provided, we based the OR and 95% confidence intervals on the broadest 95% CI conceivable for the prevalence rate.

Livingston et al. (2001) provided a RR unadjusted for age. However, since the migrant groups examined in their study were younger than the native-born population, we considered the presented RR an underestimation of the real RR and felt justified to use the unadjusted RR in our meta-analysis.

As for the meta-analyses, we calculated pooled ORs, adjusted for age and sex. First, we calculated the pooled OR for the development of dementia among all migrants in Europe (analysis 1.1). We also calculated separate pooled ORs for population surveys and for health care record studies (analysis 1.2 and analysis 1.3, respectively). Second, we calculated the pooled OR for developing dementia among African-Europeans (analysis 2.1 for all studies, analysis 2.2 for population surveys only, analysis 2.3 for African-Caribbeans and African-Surinamese). Finally, we calculated the pooled OR for developing dementia among Asian-Europeans (analysis 3.1 for all studies, analysis 3.2 for population surveys only).

The analyses were performed using SPSS version 23 and MetaWin version 2. First, a fixed effects model was used and a heterogeneity statistic, *Q*_w_, was calculated to test whether the studies could be considered to share one common population effect size. When this was not the case, a random-effect model was used. Such a model, before it estimates the overall effect estimate and its variance, weighs each study both by the variance of its individual effect size and by the between study variance. Thus, the influence of studies that produce outliers is mitigated.

## Results

After screening 5937 publications, seven articles were retained for the review and meta-analysis. Online Supplementary Fig. S1 shows the PRISMA flow-diagram. We excluded studies from which no effect estimate could be derived (*n* = 3), purely descriptive studies (*n* = 12), studies performed outside Europe or published in another language than English (*n* = 9), and studies describing associations or diagnostic instruments (*n* = 14). One article was excluded, because it used the same sample as a previous publication.

As shown in Table 1, we retrieved six prevalence studies and one incidence study (Pham et al., 2018). Five were performed in the UK, the remaining in Norway (Diaz, Kumar, & Engedal, 2015) and The Netherlands (Parlevliet et al., 2016).

**Table 1.** Studies included in meta-analysis and systematic review of risk for dementia among migrants in Europe.

First author & year	Area	Ethnicity	Study design	Age	Prevalence/incidence/incidence rate ratio	Quality assessment	Included in analysis
Adelman et al. (2011)	UK	African-Caribbean (AC)	Population-based survey	>60	AC 9.6% Reference 6.9%	High	1.1 1.2 2.1 2.2
Diaz et al. (2015)	Norway	Other income countries (OIC), High income countries (HIC)	Health care record study	>50	OIC 0.6%, HIC 1.1%, Reference 1.7%	Low	1.1 1.3
Livingston et al. (2001)	UK	African-Caribbean/African (ACA), Irish, European, Cypriot, Other	Population-based survey	>65	ACA 17.3%, Irish 3.6%, European 8.3%, Cypriot 11.3%, Other 10.0%, Reference 10.0%	Medium	1.1 1.2 2.1 2.2
McCracken et al. (1997)	UK	Black-African (BA), African-Caribbean (AC), Black-other (BO), Chinese, Asian, Other	Population-based survey	>65	BA 11.4%, AC 8.0%, BO 2.2%, Chinese 16.9%, Asian 8.3%, Other 7.1%, Reference 3%	Medium	1.1 1.2 2.1 2.2 3.1 3.2
Parlevliet et al. (2016)	The Netherlands	Turkish, Moroccan-Arabic (MA), Moroccan-Berber (MB), Asian-Surinamese (ASS), African-Surinamese (AFS)	Population-based survey	>55	Turkish 14.8%, MA 12.2%, MB 11.3%, ASS 12.6%, AFS 4.0%, Reference 3.5%	High	1.1 1.2 2.1 2.2 3.1 3.2
Pham et al. (2018)^a^	UK	Asian, Black, Mixed-other (MO)	Health care record study	>50	Asian Female 0.82 (IRR),^b^ Asian Male 0.88 (IRR),^b^ Black Female, 1.25 (IRR)^b^ Black Male, 1.28 (IRR)^b^ MO Female 0.97 (IRR),^b^ MO Male 0.86 (IRR)^b^	Low	1.1 1.3 2.1 3.1
Richards et al. (2000)	UK	African-Caribbean (AC)	Population-based survey	>65	AC 34%, Reference 2%	High	1.1 1.2 2.1 2.2

a Incidence study.

b IRR: incidence rate ratio (migrants v. natives).

Three population surveys obtained a high score for quality (Adelman et al., 2011; Parlevliet et al., 2016; Richards et al., 2000). Two studies failed to adjust the results for age differences and were considered to be of medium quality (Livingston et al., 2001; McCracken et al., 1997). Four population surveys used reputed instruments [i.e. CAMDEX-R (Roth, Huppert, Mountjou, & Tym, 1998), GMS-AGECAT (Copeland, Dewey, & Griffith-Jones, 1986), Short-CARE (Gurland, Golden, Teresi, & Challop, 1984)], but to the best of our knowledge these instruments have not been validated in minority ethnic groups in Europe.

The health care record studies (Diaz et al., 2015; Pham et al., 2018) could not be evaluated using the quality criteria for prevalence surveys. These investigations provide excellent information on the treated incidence or prevalence, but the results do not necessarily reflect the true incidence or prevalence. Since their diagnostic procedures were not standardised, we considered them to be of low quality (see online Supplementary Table S1).

Five studies defined the participants on the basis on their country of birth (Adelman et al., 2011; Diaz et al., 2015; Livingston et al., 2001; Richards et al., 2000; Parlevliet et al., 2016) and two studies divided them by self-ascribed ethnicity (McCracken et al., 1997; Pham et al., 2018). Given the history of migration to Europe, the overwhelming majority of the participants were migrants.

Since most migrants were classified as African-European or Asian-European, there was sufficient data to perform a meta-analysis on these particular populations (Table 1). No sufficient data was available to perform a meta-analysis for migrants from the Maghreb, the Middle-East or certain European countries.

**Fig. 1.**
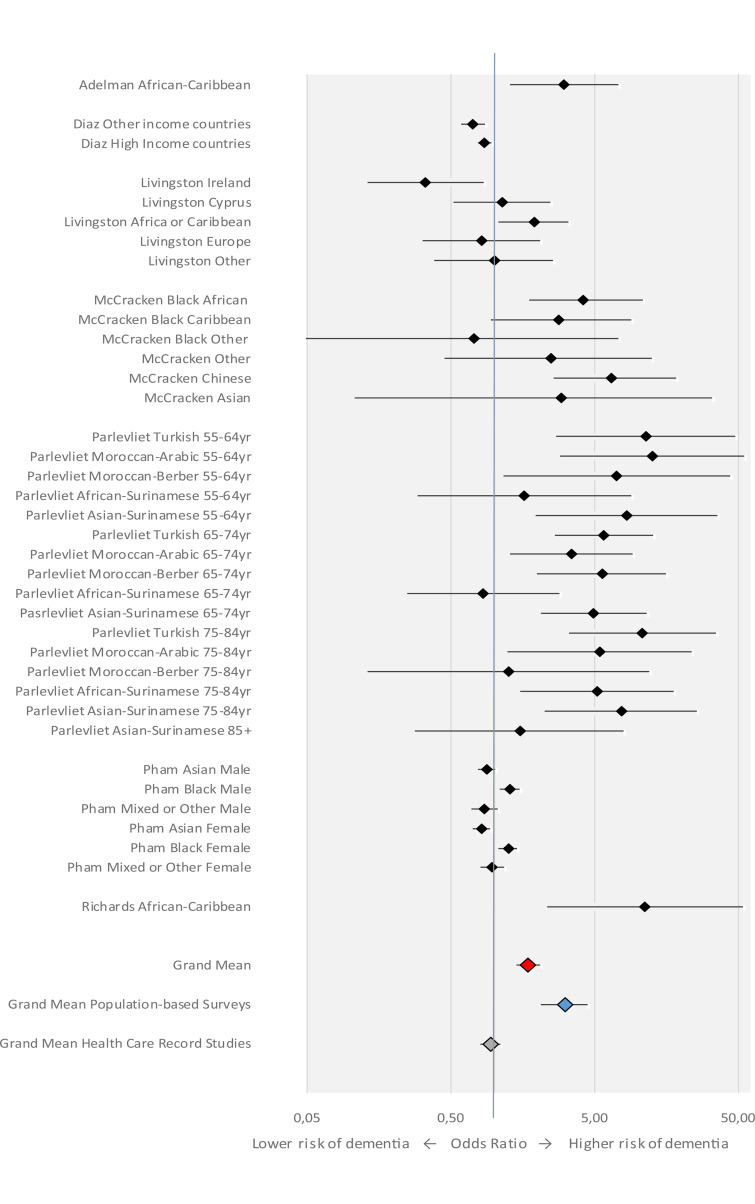
Meta-analysis of studies on risk of dementia among migrants in Europe, forest plot. The figure shows ORs for migrants *v.* the native-born, by the first author and region of origin or ethnic background of the migrant group.

The pooled OR of dementia in all migrants grouped together (Table 2; Fig. 1), as compared to the reference population, was 1.73 (95% CI 1.42–2.11). This figure increased to 3.10 (2.12–4.51) when only population-based surveys were considered (analysis 1.2), and decreased to 0.94 (0.80–1.11) when the analysis was restricted to health care record studies (analysis 1.3).

**Table 2.** Results of meta-analysis of studies examining the association between dementia and migration in Europe

Analysis	*N*^a^	Number of studies	Odds ratio^b^	95% CI	*Q*_w_^c^	*p*^d^
1.1 All migrants^e^	37	7	1.73	1.42–2.11	86.8	<0.00001
1.2 Population-based surveys^e^	29	5	3.10	2.12–4.51	26.8	0.53
1.3 Health care record studies^e^	8	2	0.94	0.80–1.11	7.6	0.37
2.1 African-Europeans^e^	11	6	1.82	1.31–2.53	15.4	0.12
2.2 Population-based surveys^f^	9	5	2.54	1.70–3.80	11.3	0.18
3.1 Asian-Europeans^e^	8	3	2.10	1.21–3.67	17.3	0.02
3.2 Population-based surveys^f^	6	2	5.36	2.78–10.31	3.3	0.66

a Number of effect estimates.

b Migrants *v*. natives.

c Measure of heterogeneity.

d *p* value for *Q*_w._

e Random-effect Model.

f Fixed-effect Model.

For African-European migrants (Table 2) the pooled OR was 1.82 (95% CI 1.31–2.53). This figure was higher (OR 2.54; 95% CI 1.70–3.80) when the analysis was restricted to population-based surveys. The pooled OR for African-Caribbeans and African-Surinamese, based on four surveys (Adelman et al., 2009; McCracken et al., 1997; Parlevliet et al., 2016; Richards et al., 2000), was 2.88 (95% CI 1.52–5.50).

The pooled OR for the Asian-European migrants (Table 2) was 2.10 (95% CI 1.21–3.67). When we limited our analysis to population-based surveys, this figure increased to 5.36 (95% CI 2.78–10.31).

Meta-analyses 2.2 and 3.2 used a fixed-effects model, because the heterogeneity was not significant. The other analyses were performed using a random-effects model. Nonetheless, significant heterogeneity remained in analysis 1.1 and 3.1.

**Fig. 2.**
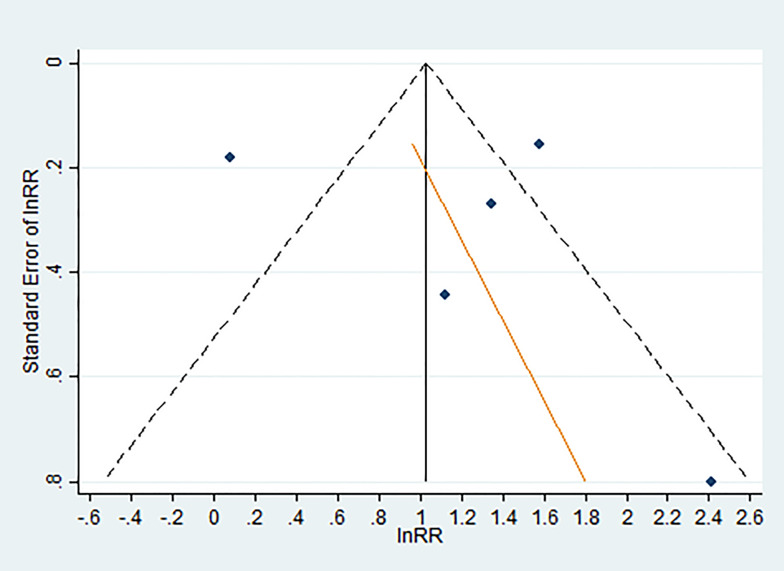
Funnel plot of studies examining the association between a history of migration to Europe and the OR (*v*. non-migrants) of dementia. The funnel plot shows:
the standard error of ln OR against the logarithmically transformed odds ratio (lnOR). In the absence of publication bias and over-dispersion (i.e. heterogeneity), the points should resemble a symmetrical inverted funnel.Egger's regression (*in red*) showing the SE plotted against the ln RR. Egger's test for funnel plot was not significant (*p* =0 .75).

A funnel plot of the population-based surveys failed to show evidence of publication bias (Fig. 2; Egger's test: *p* = 0.75).

## Discussion

To our knowledge, this is the first systematic review and meta-analysis of studies that compared the risk of dementia for migrant populations to that for the local reference populations in Europe. Seven studies have been conducted in only three countries. There was a marked discrepancy between results obtained from population-based surveys and health care record studies. Although the findings were heterogeneous, the results strongly suggest that migrants from Asia and Africa are at a significantly increased risk of developing dementia.

A strength of this meta-analysis is the high quality of some prevalence surveys. Most samples were highly representative of a well-defined target population. Parlevliet et al. (2016) used the Cross-Cultural Dementia Screening, a well-validated culturally sensitive screening instrument for dementia (Goudsmit et al., 2017), and other studies used widely accepted standardised diagnostic instruments (i.e. CAMDEX-R, GMS-AGECAT, short-CARE), which have satisfactory psychometric properties for classification of dementia.

Our meta-analysis has several limitations. First, the number of studies is small and investigations from South- or Eastern-Europe are lacking. Second, since the classification of migrants varies across studies, our division of migrants into sub-groups is necessarily somewhat arbitrary. Third, the cultural validity of most screening instruments was uncertain. Finally, the response rates of four population surveys were below 70%. The results of the survey with a higher response rate, however, also reported a significant excess of dementia among people from the Caribbean or continental Africa (Livingston et al., 2001).

Our findings for African-Europeans are in line with previous reports of increased risks of developing dementia among African-Caribbeans in the UK (Adelman et al., 2009) and African-Americans in the USA (Steenland et al., 2016; Mehta and Yeo, 2017). The estimate of the increased risk of dementia among African-Americans arrived at in the Steenland meta-analysis (RR = 1.64; 95% CI 1.35–2.00) shows remarkable similarity with the OR for African-Europeans (OR 1.82; CI 1.31–2.53), derived from our meta-analysis. Unfortunately, no comparison could be made for Asian-Europeans, as the systematic review of dementia in the USA by Mehta and Yeo (2017) considered Japanese-Americans only.

A plausible interpretation of the discrepancy between population-based surveys and health care record studies implies that migrants tend to avoid the services or remain undiagnosed. However, this is as yet uncertain, because population-based surveys might over-estimate the risk for migrants.

For an interpretation of the high risks of dementia for migrants in Europe it is important to be informed about the risk of dementia in the countries of origin. However, when one compares the pertinent risks, it is important to bear in mind that a diagnosis of dementia requires both cognitive impairment and impaired activities in daily living as a result. For people with borderline mild cognitive impairment *v.* early dementia, this distinction can be difficult to determine consistently across studies and is likely to be influenced by contextual expectations and levels of support. An older person with a given level of cognitive impairment may be more likely to show impaired activities in daily living in an unsupported urban western setting than in a more rural, traditional setting with high levels of support from extended family. Moreover, the expectations of normal function for a person of that age may be lower.

Despite this caveat, it is worthwhile to consider the World Alzheimer Report (Alzheimer's Disease International, 2009), which presents a meta-analysis of prevalence studies of dementia of all types among subjects aged 60 and over. The results were standardised for age and sex, with Western Europe as the standard population. The report shows a prevalence of 5.78% in South-Asia and 6.38% in Southeast-Asia, which is somewhat lower than the standardised prevalence for Western-Europe (7.29%). Although it was not possible to carry out a quantitative meta-analysis In African regions, due to a very small number of studies, prevalence figures were estimated using the available evidence. The standardised prevalence rates were 3.25% for Central Africa, 4.00% for Eastern Africa, 3.51% for Southern Africa and 2.07% for Western Africa. Although these figures may be biased by several factors, including selective survival, they are considerably lower than those for other parts of the world. North-Africa and the Middle-East showed a prevalence of 5.85%. The prevalence rate in the Caribbean was higher: 8.12% (see also a recent report by Davis, Baboolal, McRae, & Stewart, 2018).

Consequently, the available evidence suggests that the increased risks of dementia among migrants from Asia and Africa in Europe are not explained by correspondingly high rates in their countries of birth (see also Prince et al., 2013 and WHO, 2015). On the contrary, the prevalence rates in Asia and Africa seem to be lower than those in Europe. Which factors, then, could explain why migrants from these continents are at an even higher risk than Europeans?

The first factors to consider are atherosclerosis and atherosclerosis risk factors, which are not only associated with the risk of vascular dementia, but also with that of Alzheimer's disease (Casserly & Topol, 2004). This is important, because studies conducted in Europe have shown substantial ethnic differences in the prevalence of atherosclerosis risks factors, including metabolic syndrome and diabetes mellitus. A study in the Netherlands showed a higher prevalence of metabolic syndrome among migrants from Surinam, Turkey and Morocco (van Leijden et al., 2018). Risks of diabetes mellitus type 2 are generally increased among migrants from non-western countries, in particular among South-Asians (Barnett et al., 2006; Meeks et al., 2016). The high risk for the latter group appears to be due to a genetic susceptibility to the Western diet, which may explain the higher risk for Asians than for Africans in our study. However, it is important to note that psychosocial factors may contribute to the increased risk of atherosclerosis, because a pattern of over-eating may constitute a coping mechanism with distress.

A second factor that may play a role is a diminished cognitive reserve. The cognitive reserve hypothesis posits that higher levels of education and participation in socially or mentally stimulating activities reduce the risk of dementia. This hypothesis is supported by a large number of studies (e.g., Ott et al., 1995; Valenzuela & Sachdev, 2006), but the underlying mechanisms remain incompletely understood (Stern, 2012). Since many migrants from developing countries have a low level of education, it is very likely that this contributes to their increased risk of dementia. This can be illustrated by the following. Stern et al. (1994) followed 593 non-demented citizens of New York aged 60 years or older for over 4 years and found that individuals with less than 8 years of education had a 2.02 (95% CI 1.33–3.06) times higher risk of developing dementia compared to those with more education.

This is relevant for the present findings. In 1991 in the Netherlands, for instance, the proportions of male and female migrants from Morocco aged 50–64 years who had received no education at all were 88 and 97%, respectively. These figures were 40% and 83% for their Turkish counterparts (Central Bureau for Statistics, 1992).

However, if the relatively low prevalence rates reported for Asia and Africa are valid (Alzheimer's Disease International, 2009), not only the absolute level of education may be important, but also the ‘relative’ level of education, in comparison with that of the native-born European population. To illustrate this point, an investigation conducted in Nigeria and in Indianapolis (USA), which used the same research method at both sites, reported a significantly lower risk of dementia in Nigeria (2.3%) than among African-Americans in the USA (6.2%). This result is remarkable because levels of education and income are lower in Nigeria than in the USA (Hendrie et al., 1995).

Consequently, we should consider the possibility that one mechanism by which migration from a developing country increases the risk of dementia is a decline in social status. Interestingly, Marmot (2005) proposed that low status increases the risk of a wide range of disorders and attributed this phenomenon to over-activity of two stress pathways: the sympatho-adreno-medullary axis and the hypothalamic-pituitary-adrenal (HPA)-axis. Dysregulation of the HPA-axis contributes to the development of the metabolic syndrome. Studies of civil servants in the UK showed an inverse social gradient in the prevalence of this syndrome and in the mortality from coronary heart disease: highest risk for individuals of lowest rank (Brunner et al., 1997; Marmot, Bosma, Hemingway, Brunner, & Stansfeld, 1997). Moreover, research of non-human primates has shown an inverse relationship between rank and risk of atherosclerosis and a mediating role of cortisol (Sapolsky, 2005).

However, there may be other mechanisms whereby low status confers an increased risk of dementia. A study of mice reported that chronic social defeat stress led to a reduced expression of the endothelial cell tight junction protein Claudin-5. This impaired the integrity of the blood-brain barrier in the nucleus accumbens, with resulting infiltration of interleukin-6 into vessel walls and brain parenchyma (Menard et al., 2017). This is important because a substantial body of evidence indicates that dysfunction of the blood-brain barrier and inflammation play a role in the aetiology of vascular dementia and Alzheimer's disease (Casserly & Topol, 2004; Yamazaki & Kanekiyo, 2017).

Of note, epidemiological studies of migrants from developing countries have also shown an increased risk of schizophrenia (Selten, van der Ven, & Termorshuizen, 2020). One suggested explanation of this phenomenon is a sensitisation of the mesolimbic dopamine system due to the experience of social defeat, i.e., an inferior position or outsider status (Selten, van der Ven, Rutten, & Cantor-Graae, 2013). Thus, there may a be a parallel in the epidemiology of dementia and that of schizophrenia,

We conclude that the evidence presented here suggests that migrants from Asia and Africa are at an increased risk of developing dementia and that they are less likely to reach the services. Since migrants from these continents are also at an increased risk of schizophrenia, there may be a parallel with the epidemiology of this disorder. The findings, which may provide more insight into the aetiology or pathogenesis of dementia, need to be confirmed by further studies.

## Supplementary material

For supplementary material accompanying this paper visit https://doi.org/10.1017/S0033291720000586. click here to view supplementary material
