# Supplementary material for: Migration and dementia: a meta-analysis of epidemiological studies in Europe
Source: Psychol Med. 2020 Apr 8;51(11):1838–45. doi: 10.1017/S0033291720000586 (PMC8381287; doi:10.1017/S0033291720000586)
Supplement: Supplementary file 1 [file S0033291720000586sup.zip › S0033291720000586sup001.docx]

| First author, year of publication | Adelman  2011 | Livingston  2011 | McCracken  1997 | Parlevliet  2016 | Richards  2000 |
| --- | --- | --- | --- | --- | --- |
| Target population clearly defined? | yes | yes | yes | yes | yes |
| Probability sampling? | yes | yes | no | yes | yes |
| Response > 70%? | no | yes | no | no | no |
| Standardized assessment? | yes | yes | no | yes | yes |
| Instrument reliable? | yes | yes | yes | yes | yes |
| Instrument valid? | yes | yes | yes | yes | yes |
| Instrument culturally valid? | uncertain | uncertain | uncertain | yes | uncertain |
| Diagnosis blind to ethnicity? | yes | no | no | no | yes |
| Age-adjustment? | yes | no | no | yes | yes |
| 95% Confidence Interval? | yes | yes | yes | yes | yes |
| Total score | 8 | 7 | 4 | 8 | 8 |

**Supplementary Table 1. Quality assessment of prevalence surveys included in meta-analysis of studies of dementia among migrants in Europe. Adapted from Boyle ^1)^**

1. Boyle MH (1998). Guidelines for evaluating prevalence studies. *Evidence-Based Mental Health* 1(2), 37-40.
