# Supplementary material for: Migration and dementia: a meta-analysis of epidemiological studies in Europe
Source: Psychol Med. 2020 Apr 8;51(11):1838–45. doi: 10.1017/S0033291720000586 (PMC8381287; doi:10.1017/S0033291720000586)
Supplement: Supplementary file 1 [file S0033291720000586sup.zip › S0033291720000586sup002.docx]

Supplementary Figure 1. Flow diagram showing study selection.
