# Supplementary material for: Migration and dementia: a meta-analysis of epidemiological studies in Europe
Source: Psychol Med. 2020 Apr 8;51(11):1838–45. doi: 10.1017/S0033291720000586 (PMC8381287; doi:10.1017/S0033291720000586)
Supplement: Supplementary file 1 [file S0033291720000586sup.zip › S0033291720000586sup003.docx]

Supplementary Methods

Search strategy *Dementia and migration: a meta-analysis of epidemiological studies in*

*Europe*

PsycINFO

(DE "Dementia" OR DE "AIDS Dementia Complex" OR DE "Dementia with Lewy Bodies" OR DE "Presenile Dementia" OR DE "Semantic Dementia" OR DE "Senile Dementia" OR DE "Vascular Dementia" OR SU dementia* OR DE "Cognitive Impairment" OR SU cognit* OR SU neurocognit* OR SU "lewy body" OR SU Alzheimer) AND (TX "Turkey" OR TX "Morocco" OR TX "Suriname" OR TX "Turkish" OR TX "Moroccan" OR TX Surinam* OR TX hindustan* OR TX creol* OR TX "Northern Africa" OR TX "maghreb" OR TX "maghrib" OR "Ethnic Groups" OR DE "Immigration" OR TX emigrant* OR TX immigrant* OR TX emigrat* OR TX immigrat* OR TX migrant* OR TX foreigner* OR TX "aliens" OR TX ethnic* OR TX minorit* OR TX multiethnic* OR TX multicultur* OR TX multi-cultur* OR TX non caucas* OR TX "Caribbean Region" OR TX "african caribbean" OR TX "west-indies" OR TX "west indies" OR TX "westindies" OR TX "French speaking Africa" OR TX "French speaking African" OR TX "French-speaking Africa" OR TX "French-speaking African" OR TX "Africa South of the Sahara" OR "sub-Sahara" OR TX "subSahara" OR TX "Central Africa" OR TX "Eastern Africa" OR TX "Southern Africa" OR TX "western Africa") AND (SU "Europe" OR SU "european" OR SU "Netherlands" OR SU "Dutch" OR SU "Holland" OR SU "United Kingdom" OR SU "UK" OR SU "England" OR SU "English" OR SU "British" OR SU "Great Britain")

14-12-2018: 943 hits

Title+abstract: 28 hits

PUBMED

("Turkey"[Mesh] OR "Morocco"[Mesh] OR "Suriname"[Mesh] OR "Turkey"[tw] OR "Morocco"[tw] OR "Suriname"[tw] OR "Turkish"[tw] OR "Moroccan"[tw] OR Surinam*[tw] OR hindustan*[tw] OR creol*[tw] OR "Africa, Northern"[Mesh] OR "maghreb"[tw] OR "maghrib"[tw] OR "Minority Groups"[mesh] OR "Emigrants and Immigrants"[mesh] OR "Emigration and Immigration"[mesh] OR "Ethnic Groups"[mesh] OR emigrant*[tw] OR immigrant*[tw] OR migrant*[tw] OR foreigner*[tw] OR "aliens"[tw] OR ethnic*[tw] OR minorit*[tw] OR multiethnic*[tw] OR multicultur*[tw] OR multi-cultur*[tw] OR non caucas*[tw] OR "Caribbean Region"[Mesh] OR "african caribbean"[tw] OR "west-indies"[tw] OR "west indies"[tw] OR "westindies"[tw] OR "French speaking Africa"[tw] OR "French speaking African"[tw] OR "French-speaking Africa"[tw] OR "French-speaking African"[tw] OR "Africa South of the Sahara"[Mesh] OR "sub-Sahara"[tw] OR "subSahara"[tw] OR "Africa, Central"[Mesh] OR "Africa, Eastern"[Mesh] OR "Africa, Southern"[Mesh] OR "Africa, Western"[Mesh]) AND ("Amnesia"[Mesh] OR "Cognition Disorders"[Mesh] OR "Dementia"[Mesh] OR dementia*[tiab] OR "Lewy Body Disease"[tiab] OR Alzheimer[tiab] OR "Cognition"[Mesh] OR "Memory Disorders"[Mesh] OR neurocognit*[tiab] OR cognit*[tiab] OR "MCI"[tiab]) **AND** ("Europe"[Mesh] OR "Netherlands"[tw] OR "Dutch"[tw] OR "Holland"[tw] OR "United Kingdom"[tw] OR "UK"[tw] OR "England"[tw] OR "English"[tw] OR "British"[tw] OR "Great Britain"[tw]) NOT "Europe, Eastern"[Mesh]

14-12-2018: 1980 hits

Title+abstract: 93 hits

EMBASE

("Morocco"/ OR "Suriname"/ OR "Turkey".mp. OR "Morocco".mp. OR "Suriname".mp. OR "Turkish".mp. OR "Moroccan".mp. OR Surinam*.mp. OR hindustan*.mp. OR creol*.mp. OR exp North Africa/ OR "maghreb".mp. OR "maghrib".mp. OR exp minority group/ OR exp immigrant/ OR exp migration/ OR emigrant*.mp. OR immigrant*.mp. OR migrant*.mp. OR foreigner*.mp. OR "aliens".mp. OR ethnic*.mp. OR minorit*.mp. OR multiethnic*.mp. OR multicultur*.mp. OR multi-cultur*.mp. OR non caucas*.mp. OR exp caribbean/ OR exp "caribbean (person)"/ OR exp caribbean islands/ OR "african caribbean".mp. OR "west-indies".mp. OR "west indies".mp. OR "westindies".mp. OR "French speaking Africa".mp. OR "French speaking African".mp. OR "French-speaking Africa".mp. OR "French-speaking African".mp. **OR exp Africa/** OR exp "Africa south of the Sahara"/ OR "sub-Sahara".mp. OR "subSahara".mp. OR exp Central Africa/ OR exp South Africa/) AND (exp *"Amnesia"/ OR exp *cognitive defect/ OR exp *"Dementia"/ OR dementia*.ti. OR "Lewy Body Disease".ti. OR Alzheimer*.ti. OR exp *"Cognition"/ OR exp *"Memory Disorder"/ OR neurocognit*.ti. OR cognit*.ti. OR "MCI".ti.) **AND** (**exp western europe/**  OR "Netherlands".mp. OR "Dutch".mp. OR "Holland".mp. OR "United Kingdom".mp. OR "UK".mp. OR "England".mp. OR "English".mp. OR "British".mp. OR "Great Britain".mp.)

21-12-2018: 3014 hits

Title+abstract: 30 hits
